# “We are the ones who should make the decision” – knowledge and understanding of the rights-based approach to maternity care among women and healthcare providers

**DOI:** 10.1186/s12884-019-2189-7

**Published:** 2019-02-15

**Authors:** Yasmin Jolly, Mamuda Aminu, Florence Mgawadere, Nynke van den Broek

**Affiliations:** Centre for Maternal and Newborn Health, Liverpool School of Tropical Medicine, Pembroke Place, Liverpool, L3 5QA UK

**Keywords:** Quality of care, Respectful maternity care, Patient-provider relationship, Qualitative research

## Abstract

**Background:**

Experiences and perceptions of poor quality of care is a powerful determinant of utilisation of maternity services. With many reports of disrespect and abuse in healthcare facilities in low-resource settings, women’s and healthcare providers’ understanding and perception of disrespect and abuse are important in eliminating disrespect and abuse, but these are rarely explored together.

**Methods:**

This was a qualitative study assessing the continuum of maternity care (antenatal, intrapartum and postnatal care) at the Maternity Unit of Bwaila Hospital in Lilongwe, Malawi.

Focus group discussions (FGDs) were conducted separately for mothers attending antenatal clinic and those attending postnatal clinic. For women who accessed intrapartum care services, in-depth interviews were used. Participants were recruited purposively. Key informant interviews were conducted with healthcare providers involved in the delivery of maternal and newborn health services.

Topic guides were developed based on the seven domains of the Respectful Maternity Care (RMC) Charter. Data was transcribed verbatim, coded and analysed using the thematic framework approach.

**Results:**

A total of 8 focus group discussions and 9 in-depth interviews involving 64 women and 9 key informant interviews with health care providers were conducted.

Important themes that emerged included: the importance of a valued patient-provider relationship as determined by a good attitude and method of communication, the need for more education of women regarding the stages of pregnancy and labour, what happens at each stage and which complications could occur, the importance of a woman’s involvement in decision-making, the need to maintain confidentiality when required and the problem of insufficient human resources. Prompt and timely service was considered a priority. Neither women accessing maternity care nor trained healthcare providers providing this care were aware of the RMC Charter.

**Conclusions:**

This study has highlighted the most essential aspects of respectful maternity care from the viewpoint of both women accessing maternity care and healthcare providers. Although RMC components are in place, healthcare providers were not aware of them. There is the need to promote the RMC Charter among both women who seek care and healthcare providers.

**Electronic supplementary material:**

The online version of this article (10.1186/s12884-019-2189-7) contains supplementary material, which is available to authorized users.

## Background

Despite a 44% decline since 1990, maternal mortality remains unacceptably high with approximately 303,000 maternal deaths having occurred in 2015 [1]. In 2015, developing regions accounted for 99% of maternal deaths, with 66% occurring in sub-Saharan Africa alone [1]. Maternal death has an enormous impact on society with immediate and long-term consequences for the neonate and child [2]. Skilled birth attendance at a healthcare facility is a key strategy to reduce maternal and neonatal mortality [3]. Antenatal and postnatal care are part of the continuum of care which needs to be available to women during and after birth [4, 5].

Evidence indicates that the experiences and perceptions of poor quality of care is a powerful determinant of utilisation of maternity services in developing countries [6–8]. The White Ribbon Alliance [9] described and the rights of women in a new Respectful Maternity Care (RMC) Charter published in 2011. There is now international agreement on the need to promote women’s rights and access to safe and respectful maternal care [10].

However, disrespect and abuse are common problems in healthcare facilities [11–13]. In 2010, a review of childbirth in healthcare facilities identified seven domains of disrespect and abuse (Table 1), which constitute the universal rights of childbearing women contained in the RMC Charter. These include physical abuse, non-consented care, non-confidential care, non-dignified care (including verbal abuse), discrimination based on specific attributes, abandonment or denial of care and detention (against a woman’s will) in facilities [14]. The RMC Charter sets out the corresponding rights of women for each of the types of disrespect and abuse.

**Table 1:** The seven categories of disrespect and abuse [14] and the corresponding human rights indicators [9]

Category of Disrespect and Abuse	Corresponding Right
1. Physical abuse	Freedom from harm and ill treatment
2. Non-consented care	Right to information, informed consent and refusal, and respect for choices and preferences, including the right to companionship of choice wherever possible
3. Non-confidential care	Confidentiality, privacy
4. Non-dignified care (including verbal abuse)	Dignity, respect
5. Discrimination based on specific attributes	Equality, freedom from discrimination, equitable care
6. Abandonment or denial of care	Right to timely healthcare and to the highest attainable level of health
7. Detention in facilities	Liberty, autonomy, self-determination, and freedom from coercion

Women’s perception and experience of care is a key component of quality of maternity care [15]. Evidence suggests it influences women’s choice of place to give birth in future pregnancies [16].

The understanding and perception of both women and healthcare providers are important in eliminating disrespect and abuse, but these are rarely explored together. It is important, therefore, to draw attention to key areas of focus for interventions designed to eliminate disrespect and abuse and promote respectful maternity care, drawing from both women and healthcare providers [10, 14]. This will enable provision of context-specific and women-centred maternity services.

This study was conducted to explore knowledge and understanding of the seven domains of the RMC Charter among healthcare providers and to explore women’s perceptions regarding respectful maternity care. In particular, we sought to identify priorities identified by women themselves in order to be able to support the programmatic implementation of better quality maternity services to improve women’s uptake and experience of maternity care.

## Methods

### Study design and setting

This was qualitative study involving the use of in-depth interviews (IDI), focus group discussions (FGD) and key informant interviews (KII) at the Maternity Unit of Bwaila Hospital in Lilongwe, Malawi.

The hospital was selected for its high patient turnover, with referrals from the entire central region of Malawi. The maternity unit is one of the largest referral centres in Malawi, providing comprehensive emergency obstetric and newborn care and receives up to 14,000 births per year.

### Participants’ selection

For antenatal and postnatal care, mothers were recruited purposively from antenatal and postnatal clinics to represent the two stages of pregnancy (antenatal/postnatal) in focus group discussions. Due to the sensitive nature of intrapartum care, mothers at this stage of pregnancy were interviewed separately in an in-depth interview (Table 2).

**Table 2:** Methods of data collection and number of participants interviewed

	Type of maternity care	Method	Number of participants
Service users (Focus Group Discussions and In-Depth Interviews)	Antenatal care	5 FGDs	33
	Intrapartum care	9 IDIs	9
	Postnatal care	3 FGDs	22
Healthcare providers (Key Informant Interviews)	Antenatal care	1 KII	1
	Intrapartum care	2 KIIs	2
	Postnatal care	6 KIIs	6

Healthcare providers were selected purposively for KIIs using snowball sampling, until saturation was achieved. Participant validation conducted throughout the data collection process helped to minimise interpretive bias and significant behaviours were noted to reduce the Hawthorne effect.

The sample size was finalised upon reaching saturation in the field. The total sample consisted of 73 participants.

### Data collection

IDIs, KIIs and FGDs were conducted between May and August 2016 by the principal investigator, who was a qualified female medical doctor trained to conduct research using qualitative methods at a postgraduate degree level. All FGDs and IDIs with women receiving care were conducted in the local language (Chichewa), while interviews with healthcare providers were conducted in English.

Two topic guides (one for women and another healthcare providers) were used as flexible frameworks for data collection, with explanations provided whenever necessary. Questions were based on the Respectful Maternity Care Charter (Table 1) and the topics included; the overall treatment of women by staff, physical treatment, communication, decision-making, respect, dignity and confidentiality [9]. Both topic guides were piloted and revised before being deployed in the field (Additional file 1).

Each FGD comprised of 6–8 women. Homogenous groups were selected according to their maternity stage (antenatal/postnatal) to encourage in-depth discussions. Having considered the difficulty and sensitivity surrounding the recruitment of participants in the intrapartum stage, nine IDIs took place with women who were in their early postnatal period (≤10 days), to discuss their experiences of intrapartum care retrospectively. The service of a trained female translator was used throughout as most women communicated in the native language (Chichewa).

All interviews were recorded and transcribed verbatim. An external research assistant checked the transcripts for consistency.

### Data analysis

Transcribed material was systematically coded and analysed using the thematic framework approach [17]. Using NVivo software version 10 (QSR Int’l, 2012), themes were identified and subsequently organised according to the Respectful Maternity Care Charter categories [9].

### Ethics

Ethical approval was obtained from the Liverpool School of Tropical Medicine Ethics Committee and the National Health Science Research Committee in Malawi (1585). A written letter of permission to conduct the study was obtained from the District Health Officer. Written, informed voluntary consent was then obtained from all participants individually.

## Results

A total of 8 focus group discussions and 9 in-depth interviews involving 64 women were conducted. Also, 9 key informant interviews with health care providers were conducted (Table 2).

Emerging themes are presented according to the seven categories of the Respectful Maternity Care Charter.

### The right to be free from harm and ill treatment

Apart from one healthcare provider who mentioned (when prompted) the importance of safe intrapartum care, this right was not mentioned by women or healthcare providers as one they naturally prioritised. The majority of women and healthcare providers were familiar with the term respectful maternity care but did not really know what it meant. Two healthcare providers admitted that they had never heard of respectful maternity care.

*“Because I don’t really understand [when] you [say] ‘Respectful [maternity care]’ … I don’t think I’ve ever heard that kind of thing” - Healthcare provider*

### The right to information, informed consent and refusal, and respect for a woman’s choices and preferences, including companionship during maternity care

Participants felt that providing information in an appropriate manner played a key role in determining acceptance of healthcare providers’ advice by women, as well as giving consent for procedures, “*so that the patient should understand (the procedure)*” - Healthcare provider.

*“[It is] very important that the doctors tell [us] how everything is instead of just writing as some of [us] are illiterate.” - Woman*

In particular, women valued health education and counselling and wanted advice regarding diet plans, disease prevention and more information on HIV/AIDS and preparation for labour and birth. Several women mentioned that they would like more information about the labour process so that they could recognise when to seek medical help. In the postnatal period, women deemed it important to learn about family planning methods, how to look after their family and strongly felt that information should not be withheld in the presence of male partners. They also felt more information should be provided during the process of gaining consent.

Healthcare providers emphasised the importance of postnatal care counselling, including breastfeeding techniques, cord care and giving specific advice to HIV-positive mothers. They felt providing such information empowered and enabled women to exercise their rights in decision-making.

Communication was recognised as an important aid to rapport building;

*“ … because without communication you cannot give respectful care … without communication it means you are doing nothing.”* – Healthcare provider

Although only one healthcare provider noted the importance of listening to patients, this was frequently emphasised by women. Women also recognised it was their responsibility to ask questions and voice their concerns where necessary. Healthcare providers agreed that responding to patients’ questions and concerns was part of the process of treatment.

*“ … it’s best that there’s actually communication... [so] that you can actually tell them everything that you’re experiencing [and] they can actually help you.” - Woman*

Involving families in the decision-making process was a recurring theme amongst healthcare providers when describing respectful maternity care. Reasons for family involvement revolved around Malawi’s patriarchal culture which healthcare providers perceived as implicit and meant that men control decisions to seek care, transport to the hospital and payment for care.

### The right to privacy and confidentiality

Across the continuum of care, most participants felt that it was the healthcare provider’s duty to preserve confidentiality and that this was key to rapport building and communication. Some healthcare providers felt it was justifiable to breach confidentiality in certain circumstances, regardless of whether this damaged the patient-healthcare provider relationship. For example, seeking advice (against the mother’s wishes) from a fellow colleague. Women feared the consequences of breaching confidentiality. A minority of women used the term ‘embarrassed’ to explain how they would feel in the event of a breach of confidentiality, especially if revealing HIV test results to a person other than themselves. One woman explained that in such an event, she would consider *“not (getting) pregnant again”*.

*“It’s very important to keep the results confidential, because if the doctors tell others … That will not [be] respect [ful] to [us].” – Woman*

The issue of privacy was closely related to the theme of confidentiality and rapport building. Women did not mention lack of privacy as something of particular concern to them while healthcare providers recognised and prioritised privacy protection. Healthcare providers highlighted measures they took to protect women’s privacy during consultations with HIV-positive mothers, but made little or no mention of measures they took to protect the privacy with regard to other mothers and/or in HIV-negative women.

### The right to be treated with dignity and respect

Staff manner and behaviour was a recurring theme. Both women and healthcare providers felt it was essential to treat recipients of care with kindness. Women feel more at ease when communicating with healthcare providers who are in a “*good mood”* as this “*makes them more approachable”*. One woman believed that she must behave respectfully in order to be received respectfully. Women were mainly concerned about actually receiving maternity care (such as intrapartum care) and felt that if they were exposed during examination (lack of privacy, lack of dignity) this was less of a concern to them compared to not actually receiving any care or treatment. The theme of preserving dignity only emerged through prompting which may suggest that it was not the highest priority for women. One postnatal healthcare provider mentioned that the hospital environment contributed to the delivery of dignified, respectful maternity care. The overall commitment of healthcare providers to provide dignity and respect was also highlighted, but thought to vary with experience;

*“I feel like [the trainees] are the ones who work with [more] commitment than those … qualified [nurses]. These trainees they are not experienced … but if I [could] choose who [should help me], I would go for a trainee … because he or she is going to be fair … It’s not about being qualified, it’s about the … kind of services.” – Woman, FGD*

### The right to equality, freedom from discrimination, and equitable care

Women felt it was of vital importance that healthcare providers were non-judgemental. This was especially mentioned with regard to HIV status and also with regard to the appearance of a woman. Healthcare providers agreed with this;

*“Respectful. The first thing which comes to my mind … the client must be respected. Respected that is … to receive care … not (taking into account) age, worth, colour or religion”.*

### The right to healthcare and to the highest attainable level of health

All participants felt that human resources are key to delivering respectful maternity care. The women expressed a need for more staff in order to reduce waiting times at the hospital, while healthcare providers wanted more staff to help manage the workload. Healthcare providers believed that increasing staff numbers would result in being able to deliver aspects of respectful maternity care that they were unable to deliver given the staff shortages they faced.

*“I see 100-200 [patients] per day. So, it is very, very difficult to give respectful care as we have [discussed]... And, even in the labour ward, it is difficult; there are ten rooms and sometimes it happens that there are only four of us. How can we manage those rooms?” – Healthcare provider*

A lack of equipment and space is also seen an obstacle to delivering respectful maternity care by healthcare providers. They felt that the uninterrupted availability of medication, gowns and food for patients would enhance the maternity care experience at the hospital. It is essential to women that healthcare providers are present to provide timely care, especially during all stages of labour and birth. Punctuality was considered important with regard to administering medication and starting clinics on time.

*“It’s important that they [healthcare providers] should be there … since [they] are the ones who know whether you need help or not. For example, whether you need ‘water’ [IV fluid] or blood.” – Woman*

### The right to liberty, autonomy, self-determination, and freedom from coercion

Many women would like a doctor to make the decisions that need to be made during ante- or intrapartum care, as they were thought to be the most knowledgeable experts.

*“It’s the doctor who knows that this woman has to go for normal labour, for operation [or] for [an induction].” – Woman*

One woman said she worried about being left unattended (denied care) if she would not accept that the doctor or healthcare provider was the main decision maker. A minority of women felt that the woman herself should be the main decision maker as she understands her needs and experience better than anyone else.

*“The decision-making process should come from [me] … We are the ones experiencing the pains and actually enduring the whole process. So, doctors should only be there to advise or help [us].” – Woman*

Similarly, only a minority of healthcare providers believed that a woman should be the main decision maker during clinic appointments. Some healthcare providers said that this was dependent on the situation and staff attitude. Decisions could involve both parties but, in certain situations, would require prompt action from the healthcare provider.

*“Sometimes you find that … as a healthcare worker … [you] have to make the decision for them … when the worse comes to the worst … you use it by force...” – Healthcare provider*

## Discussion

This study explored knowledge and understanding of the seven domains of the RMC Charter among healthcare providers and explored women’s perceptions regarding respectful maternity care. Perception and experience of maternity care was explored for the continuum of care (including care during and after pregnancy and childbirth) as reported by women themselves as well as by healthcare providers providing maternity care. The principles, as set out in the RMC Charter, are those that are included in any programme aiming to improve quality of care.

The findings of this study are useful when developing strategies to improve the quality of maternal and newborn care and, thus, improve the experience of, and satisfaction with, care as well as women’s health-seeking behaviour. Many aspects of respectful maternity care were related to the importance of establishing a good rapport between the woman who seeks care and the healthcare provider. Overall, healthcare providers were seen as influential players in the implementation and practice of respectful maternity care as they had multiple roles, acting as carers, educators, advocators and decision makers.

### Staff attitude

A “*good attitude*” was deemed to be a chief determinant of the ability to provide respectful maternity care and thought to be reflected by behaviour that showed commitment and empathy and was non-judgmental. Staff commitment was valued by both women and healthcare providers. Women might choose to receive care from staff who are perceived as “*fair*” even if they are less qualified rather than from highly qualified staff who are unfriendly and disrespectful, emphasising that the quality of care received is considered just as important as the competency of the healthcare provider.

### Communication and education

Good communication was considered a vital component for enhancing rapport between women and healthcare providers. Healthcare providers listening to women, allowing women the opportunity to express concerns and ask questions as well as giving information to women in an appropriate manner were all recognized by both women and healthcare providers as being key aspects of respectful maternity care.

Educational talks and counselling sessions were regarded highly. The importance of empowering women through education and information sharing was consistent with a study by O’Donnell et al. [18]. Similarly, healthcare providers who have a fundamental role in care delivery and should receive regular updates to improve their knowledge of evidence-based care and be able to undertake appropriate training to improve their skills. Introducing professional development portfolios for maternity care providers, which are regularly reviewed by senior staff, will ensure critical self-reflection, professionalism and gradual positive change [19]. Community mobilisation for respectful maternity care rights, advocacy, dissemination of information and education regarding maternal health will also empower women and encourage them to use facility-based care when needed [20].

### Consent and decision-making

Although it is recognised that in most maternity settings healthcare providers dominate the decision-making process, this study shows that women, especially during and after pregnancy, felt that they should be the main decision makers with regard to their care. In contrast, when discussing intrapartum care, most women believed it was the healthcare provider’s duty to make decisions on their behalf. This may result from women generally feeling that they have limited knowledge regarding the physiology and process of labour and/or regarding what is required when complications occur and emergency obstetric care is needed.

Some healthcare providers felt that including a woman’s husband, partner or family in the decision-making process was necessary as, for example, traditionally the husband has the authority to agree if a woman can access facility-based care or is advised to stay at home. Although previous research suggests that elder family members especially have power to influence the decision-making process, there is emerging evidence from this study that women seek a more independent and “private” role and see themselves as the main decision makers [21].

### Privacy and confidentiality

The need to maintain confidentiality was a key emerging theme in this study. The concepts of privacy and dignity were seen as less of a priority among women in this setting, whereas healthcare providers commonly highlighted this as a matter that was very important to them. This could be explained by the finding that healthcare providers were more aware of the importance of these concepts and had sufficient knowledge regarding minimum standards of patient care needed to provide this. The finding that women themselves many still have a more limited awareness of their rights is supported by previous studies [12, 16, 20–24].

However, some of the women expressed concern about breach of confidentiality during what might be case conferencing or clinical discussions. Some particularly expressed fear of breach of confidentiality during HIV counselling and testing. Their concern may be genuine given the small communities in which they lived, where a breach of confidentiality could have severe negative consequences.

Article III of the RMC Charter provides that “Every woman has the right to privacy and confidentiality”. In this study, case conferencing was not considered a breach of confidentiality when it was done ethically, without revealing personal, identifiable details of the client.

It is of note that neither healthcare providers nor women were particularly aware of the Charter for Respectful Maternity Care. Lack of resources was seen to contribute to the absence of respectful maternity care practices in the maternity unit, similar to observations made in previous studies [18, 25].

### Strengths and limitations

There is limited published research and literature or what respectful care is considered to mean for women and whether healthcare providers and women are aware of their rights and/or the Respectful Maternity Care Charter even though this was developed and widely disseminated following its launch in 2011. This study provides new insights into an area that is little understood and seldom explored in an era of human rights advocacy. This study focused primarily on women who accessed care and healthcare providers working at a hospital in an urban area. The results are likely to be representative of similar settings on other countries. However, due to differences in cultural practices and beliefs, perceptions and experiences of those in more rural and/or more traditional areas would need further exploration.

### Implications for practice

Implementing policies to raise much greater awareness of the Respectful Maternity Care Charter at the regional, national and international levels will likely be a positive action towards improving the quality of maternal care. Conducting regular reviews of the quality of care using the Charter principles, to identify were improvements need to be made to improve the quality of care and the experiences of women who access care during and after pregnancy.

## Conclusions

This study highlights the deficiencies in knowledge regarding respectful maternity care and rights among both women accessing maternity care and trained, healthcare providers providing this care. Thus, there is a need to more proactively promote the rights of child-bearing women as outlined in the Respectful Maternity Care Charter. The role of community advocacy groups, professional association and government in promoting awareness of the concepts outlined in the Charter should also be strengthened.

## Additional file

Additional file 1: Topic guide: focus group discussions. (DOCX 26 kb)

## Abbreviations

FGD: Focus Group Discussion
IDI: In-depth interview
KII: Key Informant Interview
RMC: Respectful Maternity Care
