# Supplementary material for: “We are the ones who should make the decision” – knowledge and understanding of the rights-based approach to maternity care among women and healthcare providers
Source: BMC Pregnancy Childbirth. 2019 Feb 15;19:42. doi: 10.1186/s12884-019-2189-7 (PMC6376786; doi:10.1186/s12884-019-2189-7)
Supplement: Supplementary file 1 — Topic guide: focus group discussions. (DOCX 26 kb) [file 12884_2019_2189_MOESM1_ESM.docx]

**TOPIC GUIDE: FOCUS GROUP DISCUSSIONS**

**Introduction**

**The objectives of the FGD are:**

- Explore your perceptions of what respectful maternal care means to you
- Explore whether or not Bwaila Hospital is able to deliver services that match your idea of respectful maternal care

**Consent:**

Gain consent.

- Please feel free to interrupt me and ask me questions if you do not understand.
- You can ask for clarification at any time
- You can also withdraw from the study at any time.

**Ice breaker:**

Introduce the participants using their anonymous identification code.

**Set ground rules:**

- Explain the emphasis on confidentiality but also explain that there is no guarantee of confidentiality as members of the group may choose to breech confidentiality outside of the discussion.
- Any questions before we begin?

**Engagement Questions:**

How does being pregnant/ having given birth recently make you feel like?

**Questions to explore ideas of respectful maternal care:**

- What are your expectations when you attend your first antenatal/labour/postnatal appointment?
- How do you think you should feel when you are receiving facility-based care?
- How should staff members treat you when delivering maternal care?
- What is the role of communication in maternal care? Can you explain further?
- How should you be treated physically when receiving maternity care from a facility based hospital?
- How should you be spoken to during your contact time with a maternity care worker?
- Who do you think should make the decisions about your maternal care plan in hospital? Why?
- Can you describe your perfect antenatal clinic/labour/postnatal care?
- How would you define respectful maternal care?
- What aspect of maternity care is most important to you?
- Is there anything else you’d like to add?

**Questions to explore perceptions regarding maternity services at Bwaila Hospital:**

- Can you tell me more about past experiences you have had with maternal care services at Bwaila Hospital?
- Can you explain whether or not you feel (or have felt), that services at Bwaila Hospital have matched your expectations of care?
- Can you describe any specific experiences that stands out to you most?
- To what extent do you think that the services at Bwaila Hospital have considered and taken into account your specific needs?

**Closing comments**

- Thank you very much for taking part in this discussion today. Your contribution is highly valuable for completing my study. Is there any information that you need? If so, I will be available after the discussion if you need to ask me any questions individually. If you think of any questions later, please do not hesitate to contact me.
- If any individual is identified to have been emotionally harmed during the discussion, make a note and refer for further support if they would like to.
- Revise notes

**TOPIC GUIDE: INTERVIEWS**

**Introduction**

**The objectives of this interview are:**

- Explore your perceptions of what respectful maternal care means to you
- Explore whether or not Bwaila Hospital is able to deliver services that match your idea of respectful maternal care

**Consent:**

Gain consent.

- Please feel free to interrupt me and ask me questions if you do not understand.
- You can ask for clarification at any time
- You can also withdraw from the study at any time.

**Set ground rules:**

- Explain the emphasis on confidentiality and that all data will be anonymised.
- Any questions before we begin?

**Engagement Questions:**

What do you think is the most important role as a maternal health care provider?

**Questions to explore ideas of respectful maternal care:**

- How do you think women expect to be treated when they attend Bwaila hospital to use their maternal care services?
- How do you think the patient should feel when they receiving facility-based care?
- How should staff members treat patients when delivering maternal care?
- What is the role of communication in delivering maternal care? Can you explain further?
- How should patients be treated physically when receiving maternity care from a facility based hospital?
- How should patients be spoken to during by maternity care workers?
- Who do you think should make the decisions about a patient’s maternal care plan in hospital? Why?
- Can you describe a perfect antenatal clinic/labour/postnatal care?
- How would you define respectful maternal care?
- What aspect of maternity care is most important to you?
- Is there anything else you’d like to add?

**Questions to explore perceptions regarding maternity services at Bwaila Hospital:**

- Can you tell me of a typical experience of maternal health care a patient receives at Bwaila Hospital?
- Can you explain whether or not you feel (or have felt), that services at Bwaila Hospital matches your expectations of care delivery?
- Can you describe any specific experiences that stands out to you most?
- To what extent do you think that the services at Bwaila Hospital have considered and taken into account women’s specific needs?

**Closing comments**

- Thank you very much for agreeing to be interviews today. Your contribution is highly valuable to me in completing my study. Is there any information that you need? If you think of any questions later, please do not hesitate to contact me.
- If any individual is identified to have been emotionally harmed during the discussion, make a note and refer for further support if they would like to.
- Revise notes
